# MEK inhibitors for the treatment of non-small cell lung cancer

**DOI:** 10.1186/s13045-020-01025-7

**Published:** 2021-01-05

**Authors:** Jing Han, Yang Liu, Sen Yang, Xuan Wu, Hongle Li, Qiming Wang

**Affiliations:** 1 Department of Internal Medicine, Affiliated Cancer Hospital of Zhengzhou University, Henan Cancer Hospital, 127 Dong Ming Road, Zhengzhou, 450008 China; 2 Department of Radiotherapy, Affiliated Cancer Hospital of Zhengzhou University, Henan Cancer Hospital, 127 Dong Ming Road, Zhengzhou, 450008 China; 3 Department of Molecular Pathology, Affiliated Cancer Hospital of Zhengzhou University, Henan Cancer Hospital, 127 Dong Ming Road, Zhengzhou, 450008 China

**Keywords:** Non-small cell lung cancer, MEK inhibitors, Targeted therapy, RAS, RAF, MEK, ERK signaling pathway

## Abstract

BRAF and KRAS are two key oncogenes in the RAS/RAF/MEK/MAPK signaling pathway. Concomitant mutations in both KRAS and BRAF genes have been identified in non-small cell lung cancer (NSCLC). They lead to the proliferation, differentiation, and apoptosis of tumor cells by activating the RAS/RAF/MEK/ERK signaling pathway. To date, agents that target RAS/RAF/MEK/ERK signaling pathway have been investigated in NSCLC patients harboring BRAF mutations. BRAF and MEK inhibitors have gained approval for the treatment of patients with NSCLC. According to the reported findings, the combination of MEK inhibitors with chemotherapy, immune checkpoint inhibitors, epidermal growth factor receptor-tyrosine kinase inhibitors or BRAF inhibitors is highly significant for improving clinical efficacy and causing delay in the occurrence of drug resistance. This review summarized the existing experimental results and presented ongoing clinical studies as well. However, further researches need to be conducted to indicate how we can combine other drugs with MEK inhibitors to significantly increase therapeutic effects on patients with lung cancer.

## Introduction

Lung cancer is the most common cause of cancer-related death worldwide, with over 1.8 million lung cancer deaths annually [1]. Over the past decades, the treatment of non-small cell lung cancer (NSCLC) has changed dramatically with the development of molecular profiling, targeted therapeutic agents, and precision medicine, while the overall prognosis of lung cancer is still poor with a 5-year overall survival (OS) rate of 18% across all stages [2]. NSCLC accounts for about 80–85% of lung cancer cases and almost 70% of NSCLC patients presenting with locally advanced or metastatic disease at initial diagnosis [3]. NSCLC comprises several histologic subtypes, such as squamous cell carcinoma, adenocarcinoma, large cell or undifferentiated carcinoma. Non-squamous carcinoma (70–75%) and squamous cell carcinoma (25–30%) are two major subtypes [4]. In NSCLC somatic mutations in epidermal growth factor receptor (EGFR) and rearrangements in anaplastic lymphoma kinase gene (ALK) and ROS proto-oncogene1 (ROS1) have been validated as strong predictive biomarkers and attractive drug targets. However, the mitogen-activated protein kinase (MAPK) pathway, comprising the kinases RAS, RAF, MEK, and ERK, is also implicated in the tumorigenesis of NSCLC. Thus, MEK inhibitors’ monotherapy or combination with other targeted drugs harboring MAPK pathway become a promising strategy for NSCLC patients with B-Raf proto-oncogene (BRAF) or Kirsten rat sarcoma viral oncogene homolog (KRAS) mutations. Currently, the prevalence of BRAF mutations is 3–5% in NSCLC patients, of which BRAF V600E mutations constitute approximately 50% [5]. To date, BRAF plus MEK inhibitors have shown a remarkable survival and response rate in advanced and unresectable melanoma patients, compared with single-agent BRAF inhibition [6, 7]. Moreover, concomitant inhibition of both BRAF and MEK has been validated to overcome acquired resistance to BRAF inhibitors alone [8, 9]. Besides, the prevalence of KRAS mutations is ~ 25% and ~ 15% in Western and Asian populations with lung adenocarcinoma, respectively [10]. Although the unprecedented challenge of effective KRAS targeting is evidenced by disappointing results to date, MEK inhibitors plus other targeted agents are actively exploring the potential effect in some clinical trials right now.

The present study aimed to review researches concentrated on the effects of MEK inhibitors on NSCLC patients to facilitate the clinical management of such patients.

## Structures and functions of MEK proteins

**Fig. 1:**
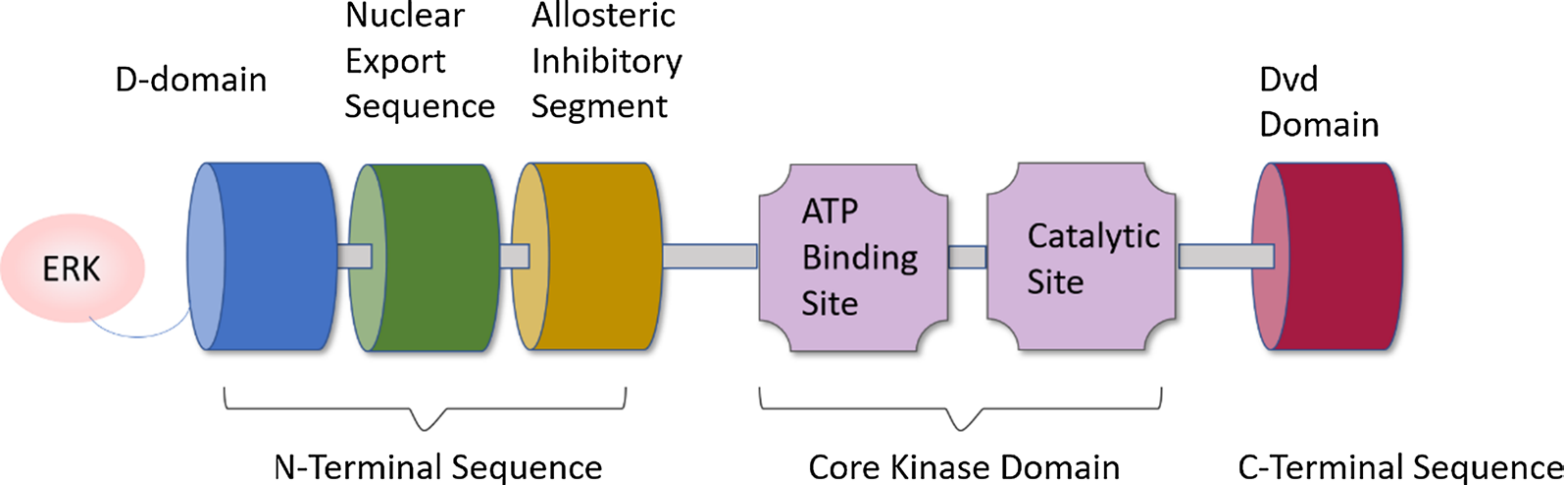
Protein structure of MEK

MEK proteins are mitogen-activated protein kinase kinase, a dual specificity Tyr/Thr protein kinase that selectively phosphorylates serine/threonine and tyrosine residues in the activation loop of ERK1 and ERK2. MEK proteins are coded by 7 different genes, among which MEK1 and MEK2 are of significance. MEK1 gene exists in human chromosome 15q22.31, and MEK2 gene exists in chromosome 9q13.3 [11]. The MEK1/2 proteins have three crucial domains (Fig. 1): a core protein kinase domain, an N-terminal domain (approximately 80 amino acids), and a shorter C-terminal region (within 30 amino acids) [11, 12]. The protein kinase domain contains the ATP site and catalytic segment; besides, a pocket structure near the ATP-binding site is an ideal target for small target agents that can change the molecule to an inactive state. The N-terminal region plays a regulatory role in signal transduction, including the D-domain (docking site) binding to the ERK substrate. Additionally, mitogen-activated protein kinase (MAPK) is localized to the cytoplasm through its specific association with the N-terminal 1–32 residues of MAPKK in unstimulated cells [13]. The C-terminal region contains the domain for versatile docking (DVD), a critical binding site for the upstream apparatus of the MAPK signaling pathway [14].

## Molecular pathways and MEK inhibitors

**Fig. 2:**
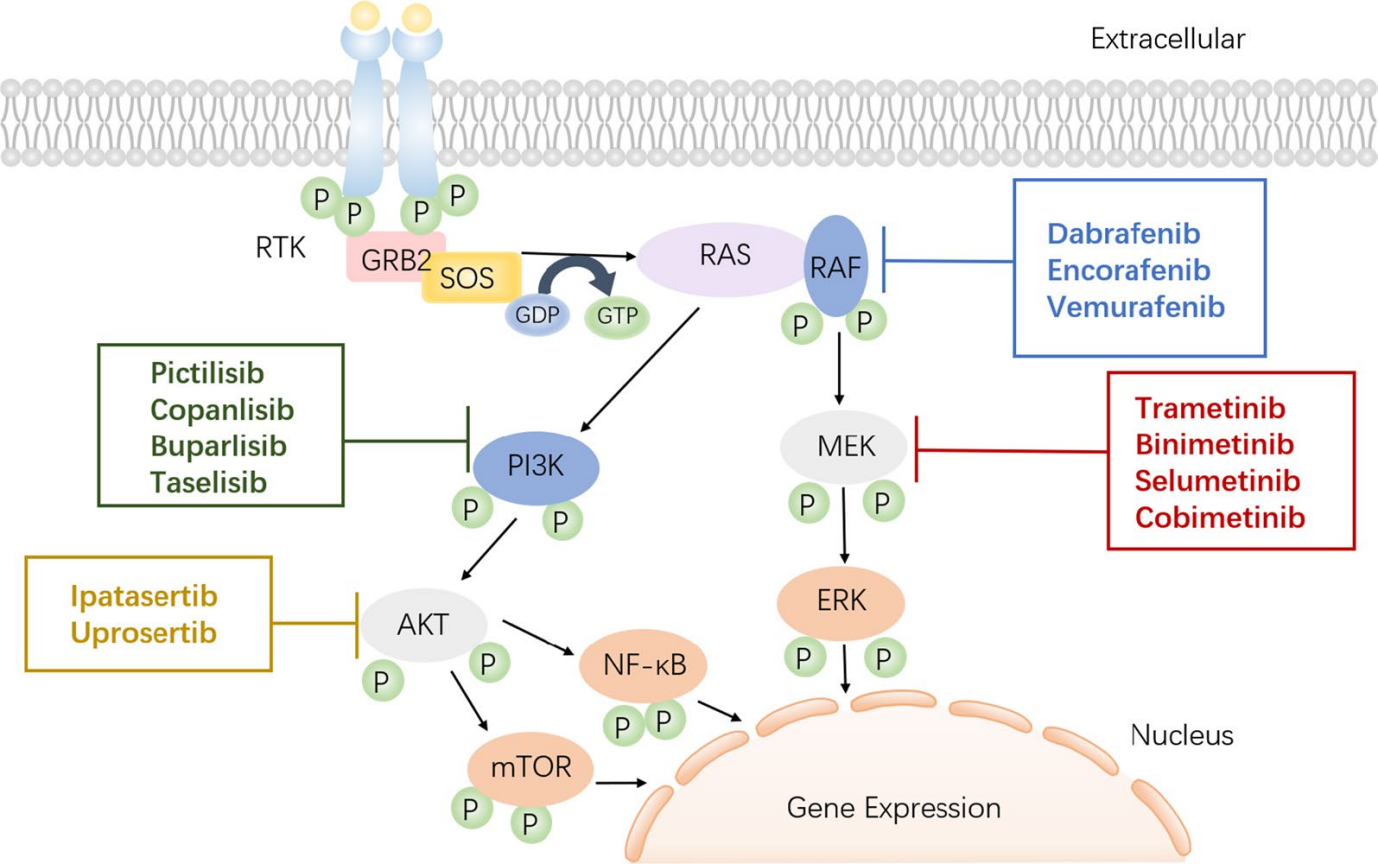
RAS/RAF/MEK/ERK signaling pathway. RTK: receptor tyrosine kinase; GRB: growth factor receptor bound protein; SOS: Son of Sevenless homolog; GDP: guanosine diphosphate; GTP: guanosine triphosphate; RAS: rat sarcoma viral oncogene; RAF: v-raf murine sarcoma viral oncogene; MEK: mitogen-activated protein kinase kinase; ERK: extracellular signal-regulated kinase; PI3K: phosphatidylinositol 3-kinase; AKT: protein kinase B; mTOR: mammalian target of rapamycin; NF-kB: nuclear factor-kB

MEK is the downstream of RAS/RAF/MEK/ERK signaling pathway, highly regulating and playing an important role in cell proliferation, differentiation, apoptosis, and stress responses [15]. It transmits mitogenic signals from outside the cell to the nucleus through multistage phosphorylation [16]. In tumor cells, certain growth factors are combined with transmembrane receptors on the cell surface, leading to the increase in RAS guanosine triphosphate-binding protein in the cell [17]. Once RAS is activated, the plasma membrane of the cell secretes and activates the downstream molecule RAF kinase, stimulates a series of protein kinases, and forms the RAS/RAF/MEK/ERK signaling pathway [18] (Fig. 2).

To date, four MEK inhibitors have been approved by the United States Food and Drug Administration (FDA), including trametinib, binimetinib, selumetinib, and cobimetinib [19–22]. They are oral, allosteric, selective, ATP-non-competitive MEK1/2 inhibitors that are not easy to produce cross-inhibition to other targets [23–27]. Notably, trametinib is the only MEK inhibitor approved for the treatment of NSCLC patients with BRAF V600E mutation in combination with dabrafenib till now (Table 1).

**Table 1:** Approval and status of MEK inhibitors in active clinical development

Drug	Developer or owner	Target	In vitro IC50 for MEK (nM)	Tumor	Approval/development status
Trametinib (GSK1120212, JTP-74057)	NOVARTIS	MEK1/2	0.7 (MEK1), 0.9 (MEK2) [24]	Melanoma, NSCLC, thyroid cancer	Approved by US FDA (05/2013)
Binimetinib (MEK162, ARRY-438162)	ARRAY BIOPHARMA INC	MEK1/2	12 [28]	Melanoma	Approved by US FDA (06/2018)
Selumetinib (AZD6244, ARRY-142886)	ASTRAZENECA	MEK1/2	14 [23]	Neurofibroma	Approved by US FDA (4/2020)
Cobimetinib (GDC-0973, XL518)	GENENTECH INC	MEK1/2	5 [29]	Melanoma	Approved by US FDA (11/2015)
Pimasertib (AS703026, MSC1936369B)	Merck KGaA	MEK1/2	30 [30]	Melanoma, ovarian cancer, pancreatic adenocarcinoma, solid tumor	I/II
Mirdametinib (PD-0325901)	Spring Works Therapeutics	MEK1/2	0.1–1000 [31]	Neurofibroma, solid tumor	I/II
Refametinib (BAY 86–9766, RDEA119)	Bayer AG	MEK1/2	19 (MEK1), 47 (MEK2) [32]	Biliary tract cancer, hepatocellular cancer, solid tumor	I/II
E6201	Eisai Co Ltd./Strategia Theraputics	MEK1/FLT3	NA	Melanoma with brain metastases, solid tumor	I
GDC-0623 (RG 7421)	Genentech, Inc.	MEK1	0.13 [33, 34]	Solid tumor	I
CH5126766(RO5126766)	Chugai Pharmaceutical Co., Roche	MEK/BRAF/CRAF	160/190/56 [35]	KRAS-mutant NSCLC, solid tumor	I
HL-085	Shanghai Kechow Pharma, Inc.	MEK1/2	1.9–10 [27]	Melanoma, NSCLC, solid tumor	I/II
SHR7390	HENGRUI MEDICINE	MEK1/2	NA	Breast neoplasm, solid tumor	I/II
TQ-B3234	CHIATAI TIANQING	MEK1/2	NA	Solid tumor	I
CS-3006	CSTONE PHARMACEUTICALS	MEK1/2	NA	Solid tumor	I
FCN-159	FOSUN PHARMA	MEK1/2	NA	NRAS-aberrant (Ia) and NRAS-mutant (Ib) melanoma	I

## Evidence for MEK monotherapy for NSCLC patients

Several trials have explored the function of single-agent MEK inhibition in early clinical development. An initial phase II study evaluated the efficacy and safety of AZD6244 versus pemetrexed as second- or third-line treatment in patients with advanced NSCLC. In this trial, 84 patients were enrolled, and 5% and 4.5% of patients achieved an objective response in AZD6244 group and pemetrexed group, respectively. However, there was no significant difference in median progression-free survival (PFS) between the two groups (90 days vs 67 days, HR:1.08, *P* = 0.79). The incidence of treatment-related serious adverse events appeared more commonly in the pemetrexed group (6.8% vs 2.5%) than in the AZD6244 group. Most frequently, toxicities were primarily dermatitis acneiform (43%), diarrhea (30%), nausea (18%), and vomiting (18%) with AZD6244 [36]. Another single-arm phase II study was conducted to test PD-0325901 in two administration schedules. This study enrolled 34 patients. Thirteen patients were administered intermittently (3 weeks on/1 week off), while 21 patients were administered adjusting schedule (5 days on/2 days off for 3 weeks, followed by 1 week off). No objective responses were observed in two schedules, while 7 patients had stable disease. Median PFS was 1.8 months (95% CI 1.5–1.9), and overall survival was 7.8 months (95% CI 4.5–13.9). The most common treatment-related toxicities (incidence in schedule A/incidence in schedule B) were diarrhea (54%/76%), fatigue (31%/48%), rash (46%/33%), vomiting (38%/33%), and nausea (38%/29%) [37]. Another phase II study evaluated the safety and efficacy of trametinib versus docetaxel for patients with KRAS-mutant NSCLC patients. In this trial, 129 were enrolled. However, there was no significant difference in median PFS in trametinib and docetaxel arm (12 weeks vs 11 weeks, HR:1.14, *P* = 0.5197) and in median OS (8 months vs not reached, HR:0.97, *P* = 0.934). Partial responses (PRs) for these two groups were 12% and 12% (*P* = 1.0000). The most frequent grade 3 or higher toxicities were primarily hypertension (9%), rash (9%), diarrhea (5%), sepsis (5%), and asthenia (5%) vs. neutropenia (35%) in trametinib and docetaxel arms, respectively. One treatment-related death occurred with trametinib and none with docetaxel [38]. An initial phase II basket trial evaluated the efficacy of selumetinib in NSCLC patients with molecular profiling. In this trial, 110 patients presented RAS/RAF mutations with KRAS mutations (24.9%), BRAF mutations (2%), HRAS and NRAS mutations (0.7%), and 10 patients were enrolled onto the selumetinib arm. However, 9 patients failed to achieve selumetinib monotherapy primary end point, with only one partial response (ORR 11%, 95% CI 0–48%), a median PFS time of 2.3 months, and median OS time of 6.5 months [39]. The results of these phase II studies indicated that MEK inhibitors’ monotherapy seemed to have poor clinical outcomes and more toxicities for NSCLC patients compared with chemotherapy alone.

## Evidences for combination of BRAF and MEK inhibitors for NSCLC patients

The combination of BRAF and MEK inhibitors has been proved to be clinically effective for NSCLC patients to date. An initial phase II trial evaluated the combination of dabrafenib and trametinib in previously treated BRAF(V600E)-mutant NSCLC patients. Fifty-seven patients were enrolled in this study. The overall response was 63.2% (95% CI 49.3–75.6%), the median PFS was 9.7 months (95% CI 6.9–19.6), and median duration of response (DOR) was 9.0 months (95% CI 6.9–18.3). Common grade 3/4 AEs were neutropenia (9%), hyponatremia (7%), and anemia (5%) [40]. Besides, the same research team developed another phase II study to assess the efficacy and safety of dabrafenib plus trametinib treatment in previously untreated patients with BRAF(V600E)-mutant metastatic NSCLC. In this study, 36 patients were enrolled and treated with first-line dabrafenib plus trametinib. The ORR was 64% (95% CI 46–79%), median DOR was 10.4 months (95%CI 8.3–17.9), and PFS was 10.9 months (95% CI 7.0–16.6). Grade 3 or 4 AEs were pyrexia (11%), alanine aminotransferase increase (11%), hypertension (11%), and vomiting (8%) [41]. The NCI-MATCH Trial Subprotocol H evaluated the combination of dabrafenib and trametinib in solid tumor patients, 5 lung adenocarcinoma patients included. One patient was progression-free at 32.5 months, and 1 patient who was considered unevaluable, with an 81% reduction in the sum of measured lesions, had a PFS of 12.7 months. Three patients had SD for 15.6, 6.6, and 3.6 months which is sought to investigate the selective BRAF inhibitor [42]. The clinical data showed the efficacy of combination of MEK and BRAF inhibitors with untreated or treated BRAF V600E-mutant metastatic NSCLC, indicating that physicians can flexibly treat patients with this targeted therapy combination in either the first-line or following chemotherapy and provide strategies to accommodate the individual patient needs.

## Evidence for combination of chemotherapy and MEK inhibitors for NSCLC patients

Chemotherapy is no longer the most efficacious treatment, and targeted agents have been rationally designed to inhibit particular mutations, leading to a more streamlined clinical trial process. Ten years ago, numerous clinical trials have concentrated on exploration of the combination of chemotherapy plus MEK inhibitors for NSCLC patients (Table 2). In the early stage, a phase II study evaluated selumetinib plus docetaxel versus docetaxel plus placebo for patients with KRAS-mutant advanced NSCLC. Forty-four and 43 patients were enrolled in selumetinib and placebo groups, respectively. The median OS was 5.2, 9.4 months (HR: 0.80, *P* = 0.21) in selumetinib and placebo group, respectively. However, the median PFS in the selumetinib group was significant longer than the placebo group (5.3 months vs. 2.1 months, HR: 0.58, *P* = 0.014). Similarly, the ORR was 37% and none (*P* < 0.0001) in selumetinib and placebo groups, respectively. Grade 3 or higher AEs occurred in 82% patients in selumetinib group and 67% patients in the placebo group (Table 3) [43]. Another phase II study of selumetinib in combination with chemotherapy was conducted in patients with advanced or metastatic non-squamous NSCLC. A total of 63 enrolled patients were randomly assigned 1:1:1 to intermittent selumetinib + chemotherapy (arm A) or continuous selumetinib + chemotherapy (arm B) or chemotherapy alone (arm C). The ORR was 35%, 62%, and 24% in arm A/B/C, respectively. Similarly, the PFS was 7.5, 6.7, 4.0 respectively. Skin and gastrointestinal adverse events were more common with the addition of selumetinib (Table 3) [44]. A phase II trial evaluating the combination of selumetinib plus docetaxel in KRAS-mutant advanced NSCLC patients also demonstrated modest improved efficacy. The retrospective analysis indicated that OS for the selumetinib + docetaxel arm vs. placebo + docetaxel arm in KRAS mutation group (MG1) and MG2 was 9.6 vs 4.4 months and 8.6 vs 7.1 months, respectively. Similarly, PFS for selumetinib and placebo groups in KRAS MG1 and MG2 was 5.7 vs 1.4 months and 4.9 vs 2.6 months, respectively. The ORR showed a numerically higher rate in MG1 compared with MG2 (46% vs 26%, respectively). Thus, for patients receiving selumetinib‏ + docetaxel and harboring KRAS G12C or G12V mutations, there were trends toward greater improvement in OS, PFS, and ORR compared with other KRAS mutations [45]. A phase 1/1b study evaluated the efficacy and safety of trametinib plus docetaxel or pemetrexed in advanced NSCLC. In this trial, 95 patients were enrolled. In trametinib plus docetaxel group, the ORR was 18% versus 24% in KRAS-WT and KRAS-mutant, respectively. In trametinib plus pemetrexed group, the ORR was 17% versus 11% in KRAS-WT and KRAS-mutant, respectively. Most common AEs were diarrhea, nausea, and fatigue (Table 3) [46]. SELECT-1 was designed to assess the efficacy and safety of selumetinib plus docetaxel in patients with KRAS-mutant locally advanced or metastatic NSCLC. In total, 510 patients were enrolled and randomized. PFS was 3.9, 1.1 months in selumetinib and placebo groups, respectively (HR:0.93, *P* = 0.44). OS was 8.7, 0.9 months (HR:1.05, *P* = 0.64), respectively. ORR was 20.1% and 13.7% in selumetinib and placebo groups, respectively. Grade 3 or higher AEs were more frequent with selumetinib group than placebo (67% vs 45%) (Table 3) [47]. However, Jacob Kaufman et al. from Duke University questioned whether other mutations are related to the response to MEK inhibition, such as the concurrent loss of tumor-suppressor genes in LKB1, which may also affect the results of the trial [48]. The SELECT-2 trial assessed the efficacy of selumetinib plus docetaxel as a second-line treatment for patients with advanced metastatic NSCLC. A total of 212 patients were randomized. There were no statistically significant improvements in PFS or OS for overall or KRAS-WT in either selumetinib or placebo group. PFS for selumetinib + docetaxel 60 mg/m^2^, selumetinib + docetaxel 75 mg/m^2^ compared with placebo + docetaxel 75 mg/m^2^ was 3.0, 4.2, and 4.3 months. The most commonly reported grade 3 or higher AE was neutropenia (Table 3) [49]. SELECT-3 trial was designed a phase I study to assess the efficacy of selumetinib in combination with platinum-doublet chemotherapy for NSCLC patients in first-line setting. Fifty-five patients were enrolled. Most frequent adverse events (AEs) were fatigue, nausea, diarrhea, and vomiting (Table 3) [50]. Another phase I study evaluated the safety and tolerability of selumetinib as a monotherapy, or in combination with docetaxel as a second-line therapy for Japanese patients with advanced NSCLC. Thirty-three patients were enrolled and 25 assigned to treatment. Grade 3 dose-limiting toxicities were febrile neutropenia and pneumonitis (Table 3) [51]. Current clinical data showed that MEK inhibitor combined with chemotherapy can improve the outcomes while some not. One possibility is that clinical benefit may occur in a specific subset of tumors that exhibits a favorable genetic of signaling environment. So effective drug candidates of MEK inhibitors and proper special patients should be detected for this combination therapy.

**Table 2:** Completed clinical trials of chemotherapy + MEK inhibitors in NSCLC

Study	Study design	Intervention	Comparation	Patient population	Patients (n)	Median OS (months)	Median PFS (months)	ORR (%)
Jänne et al. [43]	Phase2 (NCT00890825)	Selumetinib + docetaxel	Placebo + docetaxel	KRAS-mutant advanced NSCLC	87 (44 vs 43)	9.4 vs 5.2 (HR:0.8, 80% CI = 0.56–1.14, *P* = 0.21)	5.3 vs 2.1 (HR:0.58, 80%CI = 0.42–0.79, *P* = 0.014)	37% vs 0
Gandara et al. [46]	Phase1 (NCT01192165)	Trametinib + docetaxel	Trametinib + pemetrexed	NSCLC	95 (49 vs 46)	NA	KRAS wild-type:4.2 vs 5.8 KRAS-mutant type: 3.4 vs 4	KRAS wild-type:18% vs 11% KRAS-mutant type: 24% vs 17%
Jänne et al. [47]	Phase1 (NCT01933932)	Selumetinib + docetaxel	Placebo + docetaxel	KRAS-mutant NSCLC	510 (251 VS 254)	8.7 VS 7.9 (HR:1.05, 95% = 0.85–1.30, *P* = 0.64)	3.9 VS 2.8 (HR:0.93, 95%CI = 0.77–1.12, *P* = 0.44)	20.1% vs 13.7% (OR:1.61, 95%CI = 1–2.62, *P* = 0.05)
Soria et al. [49]	Phase2 (NCT01750281.)	Selumetinib + docetaxel	Placebo + docetaxel	NSCLC	212	5.7 vs 7.7 vs 11.5	3 vs 4.2 vs 4.3 (HR = 1.12,0.92)	33% vs 14% (OR:3.26, 95%CI = 1.47–7.95)
Greystoke et al. [50]	Phase1 (NCT01809210)	Selumetinib + gemcitabine/cisplatin or carboplatin	Selumetinib + pemetrexed/cisplatin or carboplatin	NSCLC	55	NA	NA	36% vs 33% vs 19% vs 13%
Seto et al. [51]	Phase1 (NCT01605916)	Selumetinib + docetaxel	Selumetinib	Solid tumor of NSCLC	25	NA	NA	NA
Melosky et al. [44]	phase2	Selumetinib + pemetrexed + cisplatin	No selumetinib	Non-squamous NSCLC	62	10 vs 10.1 vs 15.3 (HR = 1.56,1.72)(*P* = 0.31,0.2)	7.2 vs 6.9 vs 4 (HR = 0.82,0.77)(*P* = 0.56,0.44)	35% vs 62% vs 24%

NA, non-available; NSCLC, non-small cell lung cancer; ORR, objective response rate; OS, overall survival, PFS, progression-free survival; HR, hazard ratio

**Table 3:** Main grade 3 or higher adverse events

Study	Main grade 3 or higher adverse events (over 10%)
Jänne et al. [43]	Neutropenia (67%), febrile neutropenia (18%), dyspnea (2%), and asthenia (9%)
Gandara et al. [46]	Trametinib + docetaxel: anemia (16%), asthenia (4%), diarrhea (10%), dyspnea (4%), fatigue (10%), hypoalbuminemia (4%), mucosal (4%), neutropenia (22%), and stomatitis (4%). Trametinib + pemetrexed: anemia (13%), AST increased (4%), asthenia (9%), decreased appetite (4%), diarrhea (4%), dyspnea (7%), hyponatremia (15%), nausea (7%), and neutropenia (20%)
Jänne et al. [47]	Diarrhea (6%), rash (3%), nausea (1%), fatigue (2%), stomatitis (3%), edema peripheral (1%), vomiting (2%), asthenia (5%), decreased appetite (1%), dermatitis acneiform (2%), neutropenia (6%), anemia (1%) and dyspnea (2%)
Greystoke et al. [50]	Neutropenia (26%), anemia (22%), and thrombocytopenia (20%)
Seto et al. [51]	Selumetinib monotherapy: blood and lymphatic system disorders (6%), neutropenia (6%), investigations (18%), AST increase (6%), GGT increase (6%), WBC count decrease (6%), infections and infestations (6%), pneumonia (6%), gastrointestinal disorders (12%), diarrhea (6%), vomiting (6%), respiratory, thoracic and mediastinal disorders (12%), interstitial lung disease (6%), metabolism and nutrition disorders (6%) and hypoalbuminemia (6%)

## Evidence for combination of immune checkpoint inhibitors and MEK inhibitors for NSCLC patients

Immune checkpoint inhibitors (ICIs) have opened up a new era for lung cancer treatment in recent years. However, even when patients with 50% or higher positivity for PD-L1 expression are selected, overall response rates still do not exceed 31% [52, 53]. Thus, different combination treatments have been proposed. Preclinical data suggested an improved T cell activation and increased CTLA-4 expression for selumetinib and trametinib. Besides, pulsatile MEKi treatment combined with CTLA-4 blockade prolonged survival in mice-bearing tumors with mutant KRAS [54, 55]. An initial phase Ib study was conducted to investigate the safety and efficacy of cobimetinib plus atezolizumab for patients with solid tumors (n = 152), 28 NSCLC patients included. The median OS time was 13.2 months, and ORR was 18% with NSCLC. 12-month PFS and OS rates were 29% and 57% for NSCLC patients, respectively. The most common AEs were diarrhea (67%), skin rash (48%), and fatigue (40%) [56]. Another phase I/II trial evaluated immunotherapy with durvalumab and tremelimumab with continuous or intermittent administration of selumetinib in NSCLC patients. The trial is actively screening and enrolling patients, and the estimated study completion is scheduled for April 2021 [57]. Currently, the clinical data about ICI-combined MEK inhibitors are still not efficient enough to validate the most proper way to treat NSCLC. More clinical outcomes are worthy being awaited furthermore.

## Evidence for combination of EGFR tyrosine kinase inhibitors (TKIs) and MEK inhibitors for NSCLC patients

To our knowledge, acquired resistance has become a major clinical problem for advanced NSCLC patients with the increasing administration of EGFR-TKIs. The combination strategy of MEK inhibitors plus EGFR-TKIs has been proposed in certain clinical trials. Preclinical data suggested the stronger inhibitory effect of the cell proliferation of EGFR-TKIs-resistant cells for MEK inhibitors plus EGFR-TKIs [58]. A phase II study was concentrated on administration of selumetinib with and without erlotinib for KRAS-mutant and KRAS wild-type (WT) advanced NSCLC patients. Forty-one KARS-mutant and 38 KRAS-WT patients were enrolled. In KRAS-WT cohort, the median PFS was 2.1 and 2.4 months for erlotinib + selumetinib and erlotinib, respectively. Similarly, OS was 12.9 and 6.3 months, respectively. In KRAS-mutant cohort, the median PFS was 2.3 and 4.0 months for erlotinib + selumetinib and selumetinib, respectively. Similarly, OS was 21.8 and 10.5 months, respectively. In terms of safety, grade 3 and 4 toxicities were also increased in combination therapy, with diarrhea, dehydration, and fatigue all occurring in > 20% of patients [59]. TATTON was initially designed as a phase Ib trial to assess the safety and tolerability of osimertinib in combination with selumetinib, savolitinib, or durvalumab for EGFR-mutant lung cancer patients. Seventy-seven patients were enrolled in this study. The ORR was 42%, 44%, and 43% in selumetinib + osimertinib, savolitinib + osimertinib, and durvalumab + osimertinib arms, respectively. The most common AEs in selumetinib plus osimertinib group were diarrhea (75%), skin rash (58%), nausea (47%) [60]. Another phase I study evaluated the efficacy of afatinib plus selumetinib in patients with KRAS-mutant-positive solid tumors, 6 NSCLC patients included. Dose-limiting toxicities (DLTs) consisted of grade 3 diarrhea, decreased appetite, nausea/vomiting, dehydration, and mucositis. Stable disease for 221 days in a NSCLC patient was the best response [61]. In ESMO 2019 Congress, a phase I study evaluated the combination of lapatinib and trametinib for patients with KRAS-mutant solid tumors, 15 NSCLC patients included. One patient was confirmed partial response. Grade 3 AEs were diarrhea, rash, and nausea [62]. The clinical data showed that a number of trials were focused on detecting the strong rationale supporting combination therapy with MEK inhibitors for overcoming or delaying drug resistance in EGFR-mutant NSCLC. However, there are no EGFR-based combination therapies with global adoption, and therapies for patients with acquired resistance to EGFR-TKIs remain to be detected.

## Mechanisms of resistance to MEK inhibitors

RAS/RAF/MEK/ERK signaling pathway-associated inhibitors have proven to be effective in treatment of various types of cancer, but have presented drug resistance in clinical application and MEK inhibitors as well. The resistance mechanisms to MEK inhibitors have not been detected clearly to date. However, studies concentrated on metastatic melanoma and other tumors showed some underlying mechanisms expected to be overlapped. A large number of MEK-acquired drug resistance mutations have been detected, such as the acquired concurrent MEK2-Q60P mutation and BRAF V600E amplification, which conferred resistance to MEK and BRAF inhibitors [63]. MEK1^P124^ and MEK1^Q56P^ mutations were evaluated to be the mechanism of cross-resistance to PLX4720 (a selective BRAF inhibitor) and selumetinib [64]. Moreover, RAS can simultaneously induce ERK/MAPK and PI3K/AKT signaling pathways to induce drug resistance to MEK inhibitors. In preclinical studies [65–69], the combination of inhibitors, such as mTOR, PI3K, AKT/Raf, and dual inhibitors of RTK/MAPK and PI3K/AKT signaling pathways was proved to be effective to overcome drug resistance of MEK inhibitors. Besides, tumor microenvironment (TME) has been detected to play a pivotal role in promotion of the targeted therapy resistance as well [70].

## Other combined therapies and ongoing studies

As acquired resistance becomes a frequent problem for all the target agents, a number of clinical trials have been designed to evaluate the efficacy and safety of combination of two different types of targets plus MEK inhibitors, according to the probable resistance mechanisms in the former part. A preclinical experiment revealed that selumetinib combined with BEZ235 (PI3K/mTOR inhibitor) markedly enhanced their antitumor effects and inhibited the tumor growth of NCI-H1993 in gefitinib-resistant NSCLC xenograft models [71]. Other ongoing clinical trials on administration of MEK inhibitors for NSCLC patients have been summarized (Table 4). To date, a variety of MEK1/2 inhibitors have been applied for different types of cancer, including NSCLC at various stages of clinical testing. The publication of the final results of these studies is still awaited.

**Table 4:** Ongoing MEK inhibitors’ clinical trials in NSCLC

Trial NCT number	Intervention	Cancer type	Phase	Status
03170206	Binimetinib + Palbociclib	KRAS-mutant NSCLC	I/II	Recruiting
01859026	Erlotinib + Binimetinib	KRAS- or EGFR-mutant NSCLC	I/IB	Active, not recruiting
02185690	Carboplatin + Pemetrexed + Binimetinib	NSCLC	I	Active, not recruiting
02964689	Cisplatin + Pemetrexed + Binimetinib	KRAS-mutant NSCLC	I	Active, not recruiting
03581487	Durvalumab + Selumetinib + Tremelimumab	NSCLC	I/II	Recruiting
03991819	Binimetinib + Pembrolizumab	NSCLC	I	Active, not recruiting
01586624	Selumetinib + Vandetanib	NSCLC	I	Active, not recruiting
04526782	Encorafenib + Binimetinib + Docetaxel	BRAF V600E-mutant NSCLC	II	Not yet recruiting
01336634	Dabrafenib + Trametinib	BRAF V600E-mutant NSCLC	II	Active, not recruiting
04005144	Brigatinib + Binimetinib	ALK or ROS1 rearranged NSCLC	I	Recruiting
03087448	Ceritinib + Trametinib	ALK-positive NSCLC	I/II	Recruiting
01933932	Selumetinib + Docetaxel	KRAS-mutant NSCLC	III	Active, not recruiting
03600701	Atezolizumab + Cobimetinib	NSCLC	II	Recruiting
03202940	Alectinib + Cobimetinib	ALK rearranged NSCLC	IB/II	Recruiting
02642042	Trametinib + Docetaxel	KRAS-mutant stage IV NSCLC	II	Active, not recruiting
03299088	Pembrolizumab + Trametinib	KRAS-mutant NSCLC	I	Recruiting
03516214	EGF816 + Trametinib	EGFR-mutant NSCLC	I	Recruiting
03225664	Trametinib + Pembrolizumab	NSCLC	I/II	Recruiting
01750281	Selumetinib + Docetaxel	NSCLC	II	Active, not recruiting
02664935	National Lung Matrix Trial: AZD4547/Vistusertib/Palbociclib/ Crizotinib/ Selumetinib/Docetaxel/AZD5363/ Osimertinib/ Durvalumab/ Sitravatinib/AZD6738	NSCLC	II	Recruiting
03990077	HL-085 + Docetaxel	KRAS-mutant NSCLC	I	Not yet recruiting
01912625	Trametinib + Carboplatin + Paclitaxel + Radiation Therapy	NSCLC	I	Active, not recruiting

Data source: www.clinicaltrials.gov, cutoff data: October 24, 2020

## Conclusions/expectations

The functions of EGFR-TKIs, checkpoint inhibitors, and traditional chemotherapy have been widely studied in NSCLC patients, while the role of MEK inhibitors in the treatment of lung cancer has not been clearly described. A number of clinical trials explored the clinical application of MEK inhibitors, and combination therapy has demonstrated promising outcomes. The brief summarization of MEK inhibitors in the selected clinical trials with NSCLC can be found in Table 5.

**Table 5:** MEK inhibitors in clinical trials

Study	Phase	MEK inhibitors	Drug therapy
Hainsworth et al. [36]	Phase II	Selumetinib	MEKi
Haura et al. [37]	Phase II	Mirdametinib	MEKi
Blumenschein et al. [38]	Phase II	Trametinib	MEKi
Lopez-Chavez et al. [39]	Phase II	Selumetinib	MEKi
Planchard et al. [40]	Phase II	Trametinib	MEKi + BRAFi
Planchard et al. [41]	Phase II	Trametinib	MEKi + BRAFi
Salama et al. [42]	Phase II	Trametinib	MEKi + BRAFi
Jänne et al. [43]	Phase II	Selumetinib	MEKi + CT
Gandara et al. [46]	Phase I/ Ib	Trametinib	MEKi + CT
Jänne et al. [47]	Phase III	Selumetinib	MEKi + CT
Soria et al. [49]	Phase II	Selumetinib	MEKi + CT
Greystoke et al. [50]	Phase I	Selumetinib	MEKi + CT
Seto et al. [51]	Phase I	Selumetinib	MEKi + CT
Melosky et al. [44]	Phase II	Selumetinib	MEKi + CT
Hellmann et al. [56]	Phase Ib	Cobimetinib	MEKi + ICI
Gaudreau et al. [57]	Phase I/II	Trametinib	MEKi + ICI
Carter et al. [59]	Phase Ib	Selumetinib	MEKi + EGFR-TKI
Oxnard et al. [60]	Phase Ib	Selumetinib	MEKi + EGFR-TKI

MEKi, MEK inhibitors, BRAFi, BRAF inhibitors, CT, chemotherapy, ICI, immune checkpoint inhibitors, EGFR-TKI, epidermal growth factor receptor tyrosine kinase inhibitors

At the early stage, MEK inhibitors’ monotherapy had been detected a lot but seemed not to be effective for NSCLC patients for its poor efficacy and higher toxicities. No matter compared with pemetrexed or docetaxel, no significant difference in median PFS or OS was observed and dermatitis acneiform, hypertension, and diarrhea toxicities were more common [36–39].

MEK inhibitors in combination with BRAF inhibitors as a treatment demonstrated an improved efficacy for NSCLC patients. Currently, trametinib combined with dabrafenib has been the only therapy approved by the United States Food and Drug Administration (FDA) and European Medicines Agency (EMA) for the treatment of BRAF V600E-mutant NSCLC patients, which has been written into the National Comprehensive Cancer Network (NCCN) guidelines as well. The phase II trials in previously treated and untreated BRAF V600E-mutant NSCLC demonstrated that PFS and OS were longer and ORR was higher, which were much better than the outcomes of single-agent BRAF inhibitor in the previous study [40, 41, 72]. According to these trials, NCCN guidelines recommend that dabrafenib combined with trametinib be the first-line and subsequent therapy for BRAF V600E mutation-positive NSCLC patients. However, the challenge with this combination is the emergence of drug resistance and no effective treatment strategy to overcome it yet. Another challenge in targeted therapy for non-V600E mutation patients is still lacking.

Chemotherapy plus MEK inhibitors have showed obscure clinical outcomes to date. Some trials demonstrated that this combination therapy had the trend of longer PFS, OS, and higher ORR, but with no significant difference, especially in the SELECT series trials [47, 49, 50]. Other trails [44–46] tried to do some exploration in subgroup NSCLC patients, such as KRAS-mutant and KRAS-WT patients. Regrettably, different chemotherapy drugs seemed to influence the outcomes as well. A phase 1/1b study showed that ORR was 18% versus 24% in KRAS-WT and KRAS-mutant patients in trametinib plus docetaxel group, while ORR was 17% versus 11% KRAS-WT and KRAS-mutant patients in trametinib plus pemetrexed group [46]. Chemotherapy applied concurrently with MEK inhibitors requires further specific validation including the different chemotherapy agents, KRAS or other gene mutations and different MEK inhibitors before this combination strategy can become a standard treatment option for NSCLC patients.

Based on the preclinical studies, MEK inhibitors could improved T cell activation, conditioned the tumor microenvironment to facilitate improved response to anti-CTLA-4 treatment and prolonged survival in KRAS-mutant mice in combination with CTLA-4 blockade [54, 55]. However, the current relevant clinical trials of ICIs plus MEK inhibitors were not sufficient to draw the conclusion yet. Since only a phase Ib study [56] investigated the safety and efficacy of cobimetinib plus atezolizumab in a single arm for few NSCLC patients and several PD-1/L1 inhibitors plus MEK inhibitors clinical trials [57] (Table 4) are still ongoing, the finial clinical outcomes are worthy being looking forward to furthermore.

Although targeted therapy has dramatically changed our approach to treating NSCLC, the emergency of drug resistance and the lack of effective treatments to some special target such as KRAS still affect the prognosis of NSCLC patients. Preclinical data showed that MEK inhibitors plus EGFR-TKIs could inhibit cell proliferation significantly of EGFR-TKIs-resistant cells, while similar clinical trials have not been designed yet [58]. Current clinical trials [59–62] focused on EGFR-TKIs, including erlotinib, osimertinib, and afatinib, in combination with MEK inhibitors appearing somewhat illusory for OS, PFS or ORR. These outcomes seemed not to be improved under this strategy and more obvious toxicities were revealed. Further researches should be designed more on the administration way to combine two or three drugs together to optimize the therapeutic effect in appropriate subset patients.

In addition to the emerging drugs and clinical studies mentioned above, there are still many more new treatment combinations that have conducted in early stages of clinical development. Novel combination drugs can be broadly classified as BRAF inhibitors, EGFR-TKIs, multi-target tyrosine kinase inhibitors, CDK4/6 inhibitors, ALK inhibitors, platin-based chemotherapy, and ICIs. Additionally, many treatment combinations being explored in early-stage clinical studies, such as PI3K and AKT inhibitors should be further detected in a more rational way with MEK inhibitors in human bodies [73–77] (Fig. 2). The preclinical data indicated that the combined therapy of MEK and PI3K inhibitors has presented promising outcomes for NSCLC patients with the acquired resistance to EGFR-TKIs [78], but more clinical effects should be validated in the future. Overall, there seems to be hope on the horizon for NSCLC patients administrated with MEK inhibitors combined with other promising agents to improve patient outcomes finally.

## Data Availability

Not applicable as no datasets were generated or analyzed.

## Abbreviations

NSCLC: Non-small cell lung cancer
RTK: Receptor tyrosine kinase
GRB: Growth factor receptor bound protein
SOS: Son of Sevenless homolog
GDP: Guanosine diphosphate
GTP: Guanosine triphosphate
RAS: Rat sarcoma viral oncogene
RAF: V-raf murine sarcoma viral oncogene
MEK: Mitogen-activated protein kinase kinase
ERK: Extracellular signal-regulated kinase
PI3K: Phosphatidylinositol 3-kinase
AKT: Protein kinase B
mTOR: Mammalian target of rapamycin
NF-kB: Nuclear factor-kB
NA: Non-available
ORR: Objective response rate
OS: Overall survival
PFS: Progression-free survival
HR: Hazard ratio
MEKi: MEK inhibitors
BRAFi: BRAF inhibitors
CT: Chemotherapy
ICI: Immune checkpoint inhibitors
EGFR-TKI: Epidermal growth factor receptor tyrosine kinase inhibitors
DLTs: Dose-limiting toxicities
NCCN: National Comprehensive Cancer Network
FDA: The United States Food and Drug Administration
EMA: European Medicines Agency
